# Nuclear Receptors in Nonalcoholic Fatty Liver Disease

**DOI:** 10.1155/2012/139875

**Published:** 2011-12-08

**Authors:** Jorge A. López-Velázquez, Luis D. Carrillo-Córdova, Norberto C. Chávez-Tapia, Misael Uribe, Nahum Méndez-Sánchez

**Affiliations:** Liver Research Unit, Medica Sur Clinic & Foundation, Puente de Piedra 150, Colonia Toriello Guerra, 14050 Tlalpan, Mexico City, Mexico

## Abstract

Nuclear receptors comprise a superfamily of ligand-activated transcription factors that are involved in important aspects of hepatic physiology and pathophysiology. There are about 48 nuclear receptors in the human. These nuclear receptors are regulators of many hepatic processes including hepatic lipid and glucose metabolism, bile acid homeostasis, drug detoxification, inflammation, regeneration, fibrosis, and tumor formation. Some of these receptors are sensitive to the levels of molecules that control lipid metabolism including fatty acids, oxysterols, and lipophilic molecules. These receptors direct such molecules to the transcriptional networks and may play roles in the pathogenesis and treatment of nonalcoholic fatty liver disease. Understanding the mechanisms underlying the involvement of nuclear receptors in the pathogenesis of nonalcoholic fatty liver disease may offer targets for the development of new treatments for this liver disease.

## 1. Introduction

 Liver diseases are a serious problem throughout the world. In Mexico, since 2000, cirrhosis and other chronic liver diseases have become among the main causes of mortality [1]. The incidence and prevalence of liver diseases are increasing along with changes in lifestyle and population aging, and these diseases were responsible for 20,941 deaths in 2007 [2].

In Mexico, the incidence of metabolic syndrome is also increasing. The metabolic syndrome has recently been associated with nonalcoholic fatty liver disease (NAFLD), and about 90% of patients with NAFLD have more than one feature of the metabolic syndrome [3]. The severity of NAFLD is one factor contributing to the development of nonalcoholic steatohepatitis (NASH), cirrhosis, and hepatocellular carcinoma [4, 5]. The growing obesity epidemic requires a better understanding of the genetic networks and signal transduction pathways that regulate the pathogenesis of these conditions. A clear definition of the mechanisms responsible for metabolic control may provide new knowledge for the development of new drugs, with novel mechanisms of action, for the treatment of chronic liver diseases.

The ability of individual nuclear receptors (NRs) to regulate multiple genetic networks in different tissues and their own ligands may represent a new class of potential drugs targets. To elucidate the challenges involved in developing such drugs, this paper focuses on the role of hepatic NRs in lipid metabolism and the possible effects on the physiopathology of NAFLD.

## 2. Nonalcoholic Fatty Liver Disease

NAFLD is defined by the accumulation of triglycerides in the form of droplets (micro- and macrovesicles) within hepatocytes [6]. The mechanism involves impaired insulin regulation, which affects fat and glucose metabolism (intermediary metabolism) in the liver, skeletal muscle, and adipose tissue, a condition known as insulin resistance. Insulin resistance increases free fatty acids and hepatic *de novo* lipogenesis, causes dysfunction in fatty acid oxidation, and alters very-low-density lipoprotein (VLDL) triglyceride export [7].

NAFLD is associated with insulin resistance, obesity, and a lifestyle characterized by physical inactivity and an unlimited supply of high-fat foods. However, more recent studies have proposed that not all individuals with NAFLD develop insulin resistance before the presence of a fatty liver [3, 8].

NAFLD is a cluster of metabolic, histological, and molecular disorders characterized by liver injury [9]. The purpose of this paper is to describe the complex working of NRs and their role in the hepatic accumulation of fat independent of excessive alcohol consumption.

NRs are ligand-activated transcription factors that have a broad range of metabolic, detoxifying, and regulatory functions. NRs are sensitive to the levels of many natural and synthetic ligands including hormones, biomolecules (lipids), vitamins, bile acids, metabolites, drugs, and xenobiotic toxins. In addition to their functions at the hepatic level, NRs also control hepatic inflammation, regeneration, fibrosis, and tumor formation [10]. These functions can be understood through a complex transcriptional network that allows them to maintain cellular nutrient homeostasis, to protect against toxins by limiting their uptake and facilitating their metabolism and excretion, and to play a role in several key steps in inflammation and fibrosis [11].

New knowledge about the functions of NRs helps clarify the pathogenesis and pathophysiology of a wide spectrum of hepatic disorders (see Table 1).

## 3. Nuclear Receptor Structure

**Figure 1:**
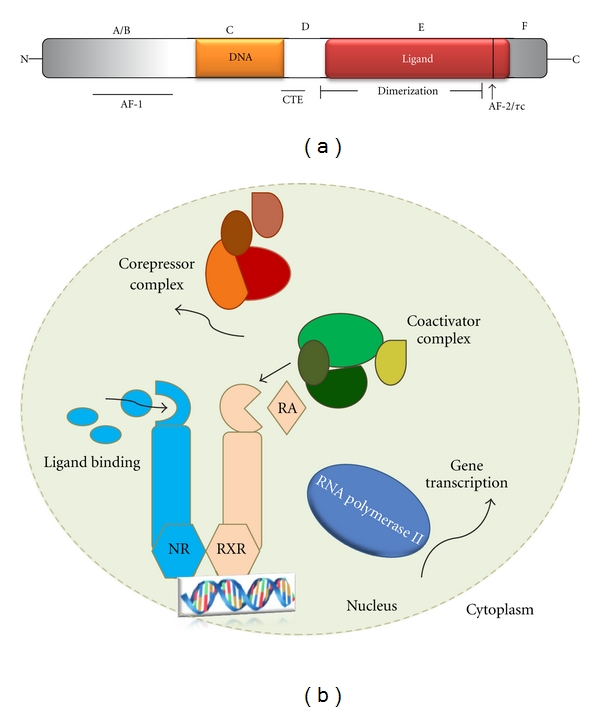
(a) Schematic representation of a typical nuclear receptor. Nuclear receptors may be divided into five regions based on structural and functional similarities (denoted A, B, C, D, E, and F). Regions C and E contain the conserved DNA-binding domains (DBDs) and ligand-binding domains (LBDs) that are the signature of this superfamily. In addition, the constitutive transport element (CTE) is a dimerization region within the LBD and two transactivation domains (denoted AF-1 and AF-2/*τ*c). A second dimerization domain (not shown) exists in the DBD and is required for heterodimerization of receptors on response elements. (b) NR function. Ligand binding to NRs triggers changes in their conformation leading to the dissociation of corepressors and the recruitment of coactivators. After this exchange of coregulators, RNA polymerase II is recruited and mRNA transcription is initiated. Most NRs bind to their DNA response elements in a sequence-specific manner as dimers, functioning either as homodimers or as heterodimers with the RXR. RA: retinoic acid. Modified from [13, 94].

The NRs are characterized by a central DNA-binding domain, which targets the receptor to specific DNA sequences known as hormone-response elements. The DNA-binding domain comprises two highly conserved zinc fingers that isolate the nuclear receptors from other DNA-binding proteins. The C-terminal half of the receptor encompasses the ligand-binding domain, which possesses the essential property of ligand recognition and ensures both specificity and selectivity of the physiological response [12, 13]. The predominant role of these receptors is the transcriptional regulation of enzymes and other proteins involved in energy homeostasis (Figure 1(a)).

## 4. Action Mode of Nuclear Receptors

NRs act in three steps [14]: repression, derepression, and transcription activation. Repression is characteristic of the apo-NR, which recruits a corepressor complex with histone deacetylase activity. Derepression occurs following ligand binding, which dissociates this complex and recruits the first coactivator complex, with histone acetyltransferase activity, and causes chromatin decondensation, which is believed to be necessary, but not sufficient, for activation of the target gene. In the third step, transcription activation, the histone acetyltransferase complex dissociates to cause the assembly of a second coactivator, which can establish contact with the basal transcriptional machinery to activate the target gene [15] (Figure 1(b)).

Coactivators are molecules recruited by ligand-bound activated NRs (or other DNA-binding transcription factors) that increase gene expression. Coactivators contribute to the transcriptional process through a diverse array of enzymatic activities such as acetylation, methylation, ubiquitination, and phosphorylation, or as chromatin remodelers [16].

The result is the modulation of the expression of a wide array of physiologically important groups of genes involved in diverse pathological processes including cancer, inherited genetic diseases, metabolic disorders, and inflammation.

In contrast to the coactivator function, corepressors interact with NRs that are not bound to the ligand and repress transcription. Corepressor-associated proteins such as histone deacetylases enforce a local chromatin environment that opposes the transcription-promoting activities of coactivators [17].

## 5. Nuclear Receptors in the Liver

The hepatocyte is responsible for processes involved in providing for many of the body's metabolic needs, including the synthesis and control of the pathways involved in the metabolism of cholesterol, fatty acids, carbohydrates, amino acids, serum proteins, and bile acids, and the detoxification of drugs and xenobiotics.

The hepatocyte employs multiple levels of regulation to perform its functions and possesses self-protective processes to avoid self-destruction. Some members of the NR superfamily provide hepatic mechanisms for self-regulation in hepatocytes [18].

Gene regulation by NRs is more complex than simply the presence of a potential DNA recognition sequence in a promoter. Rather, it is a complex and multilayered process that involves competition between agonists and antagonists, heterodimerization, coregulator recruitment, and NR protein modification.

The NR family comprises 48 family members and is the largest group of transcriptional regulators in the human. Because some NRs participate in the control of hepatic homeostasis, they may provide a new therapeutic target for the treatment of liver diseases such as NAFLD [19].

### 5.1. Liver X Receptor

The transcriptional factor liver X receptor (LXR) is involved in cholesterol metabolism. The LXR gene encodes two distinct products, LXR*α* and LXR*β*, each with diverse patterns of expression but similar target DNA-binding elements and ligands. The human LXR*α* gene is located on chromosome 11p11.2, and the human LXR*β* gene is located on chromosome 19q13.3. We will focus on LXR*α* because of its high expression in the liver, although it is also expressed at lower levels in the kidney, intestine, lung, fat, adrenal, spleen, and macrophages [20, 21]. The ligands for LXR are oxysterols. Once activated, LXR induces the expression of a cluster of genes that function in lipid metabolism; these functions are cholesterol absorption, efflux, transport, and excretion [22–24]. Besides its metabolic role, LXRs also modulate immune and inflammatory responses in macrophages [25]. 

Like most other nuclear receptors, LXR forms heterodimers with the retinoid X receptor (RXR) within the nucleus. Binding of the RXR to LXR leads to the formation of a complex with corepressors such as silencing mediator of retinoic acid, thyroid hormone receptor, and nuclear corepressor [26].

In the absence of a ligand, these corepressor interactions are maintained and the transcriptional activity of target genes is suppressed. Binding of a ligand to LXR causes a conformational change that facilitates inactivation of the corepressor complex and the transcription of target genes [27].

**Figure 2:**
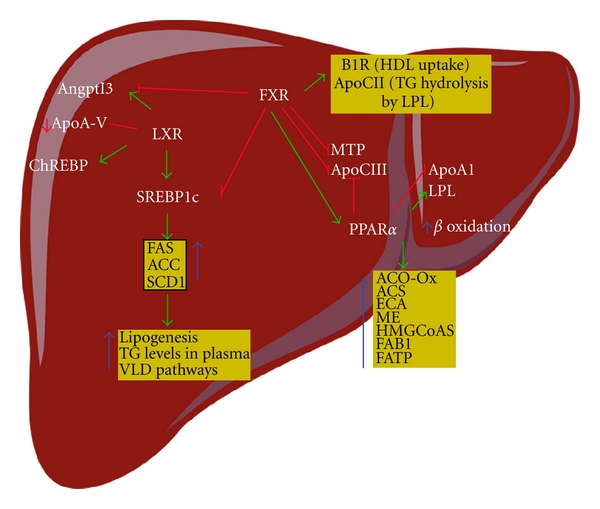
NRs as central regulators of hepatic lipid metabolism. Oxysterols activate the LXR, whereas bile acids (BA) stimulate SHP expression through the FXR (not shown). The LXR activates SREBP-1c and induces *de novo* fatty acid (FA) synthesis and hypertriglyceridemia by activating FAS, ACC, SCD1, and ChREBP (a glucose-sensitive transcription factor that promotes the hepatic conversion of carbohydrates into lipids). Several important proteins that could mediate the LXR-mediated hypertriglyceridemic effect are regulated. One protein is angiopoietin-like protein 3 (Angptl3), a liver-secreted protein that increases both plasma triglyceride level by inhibiting LPL activity in different tissues and free fatty acid level by activating lipolysis in adipocytes. LXR activation increases the expression of Angptl3 and LPL and downregulates apoA-V expression. Activation of the FXR leads to the repression of hepatic lipogenesis by reducing the expression of SREBP-1c. By increasing the expression of PPAR*α*, the FXR also promotes FFA catabolism via *β*-oxidation, which induces ACO-OX, ACS, ECA, HMG-CoAS, FAB1, and FATP. By repressing the expression of MTP, an enzyme that controls VLDL assembly, the FXR reduces VLDL production. Activation of the FXR increases TG clearance by promoting LPL activity, via induction of ApoC-II and B1R. Activation of the FXR also reduces TG clearance by decreasing the expression of ApoC-III and Angptl3, two LPL inhibitors. PPAR*α* can be activated by FXR and fibrates (not shown). PPAR activation leads to *β*-oxidation, which induces ACO-Ox, ACS, ECA, HMG-CoAS, FAB1, and FATP. Others genes are regulated. For example, LPL, which is involved in the degradation of TG, is activated, and APOA1 and APOCIII are both downregulated. The activation pathways are shown by green arrows, inhibitory pathways by red lines, and inhibited activation pathways by broken green arrows. Angptl3: angiopoietin-like protein 3; ACC: acetyl-CoA carboxylase; Apo: apolipoprotein; ChREBP: carbohydrate response element-binding protein; FAS: fatty acid synthase; FATP: fatty acid transport protein; FXR: farnesoid X receptor; LPL: lipoprotein lipase; LXR: liver X receptor; MTP: microsomal triglyceride transfer protein; PPAR: peroxisome proliferator-activated receptor; SCD1: stearoyl-coenzyme A desaturase 1; SREBP-1c: sterol regulatory element-binding protein-1c; TG: triglyceride. Arrows and stop bars indicate positive regulation or activation and negative regulation or repression, respectively.

LXR is a key regulator of whole-body lipid and bile acid metabolism [20, 28] (Figure 2). LXR regulates a cluster of genes that participate in the transport of excess cholesterol in the form of high-density lipoprotein (HDL) from peripheral tissue to the liver—a process called reverse cholesterol transport. *In vivo* activation of LXR with a synthetic, high-affinity ligand increases the HDL level and net cholesterol secretion [29]. LXR positively regulates several enzymes involved in lipoprotein metabolism including lipoprotein lipase (LPL), human cholesteryl ester transport protein, and the phospholipid transfer protein [30]. LXR also regulates the crucial bile acid enzyme CYP7A1. In rodents, this enzyme contains an LXR response element that is upregulated in response to excess cholesterol in the diet. The enzymatic activation and conversion of cholesterol to bile acids is one mechanism for handling excess dietary cholesterol [31–33].

In addition to its ability to modulate cholesterol and bile acid metabolism, LXR is also a key regulator of hepatic lipogenesis. Its lipogenic activity results from the upregulation of the master regulator of hepatic lipogenesis sterol regulatory element-binding protein-c (SREBP-c) and from the induction of fatty acid synthase, acyl coenzyme A carboxylase, and stearoyl CoA desaturase 1, all leading to increased hepatic lipid levels [34, 35], one of the etiological agents in the pathogenesis of NAFLD. Moreover, LXR induces the carbohydrate-response element-binding protein, ChREBP [36]. ChREBP is a target gene of LXR and is a glucose-sensitive transcription factor that promotes the hepatic conversion of carbohydrates into lipids. Several important proteins might mediate the LXR-mediated hypertriglyceridemic effect. These include angiopoietin-like protein 3 (Angptl3) [37], a liver-secreted protein that increases the concentrations of both plasma triglycerides by inhibiting LPL activity in different tissues and free fatty acids by activating lipolysis in adipocytes and/or apoA-V. LXR activation increases Angptl3 expression and downregulates apoA-V expression [38]. The second “hit” in NAFLD is related to the proinflammatory molecules, whose expression is repressed by LXR. These include inducible nitric oxide synthase, cyclooxygenase 2, interleukin-6 (IL-6), IL-1*β*, chemokine monocyte chemoattractant protein-1, and chemokine monocyte chemoattractant protein-3 [39].

LXR-activated pathways play central roles in whole-body lipid metabolism by regulating multiple pathways in liver cells. Further investigation into the effects of synthetic LXR-specific agonists and/or antagonists may provide new therapeutic tools for the treatment of NAFLD.

### 5.2. Peroxisome Proliferator-Activated Receptors

NAFLD appears to be a link between insulin resistance and obesity. Several recent studies have shown that a family of transcription factors, named the peroxisome-proliferator-activated receptors (PPARs), improve several of the metabolic abnormalities associated with insulin resistance and impaired fat metabolism [40].

The PPARs are nuclear hormone receptors. Three isotypes have been identified in humans: PPAR*α*, PPAR*β*/*δ*, and PPAR*γ* [41]. These receptors exhibit different tissue distribution and functions and, to some extent, different ligand specificities. PPAR*α* is highly expressed in the liver, brown adipose tissue, heart, skeletal muscle, kidney, and at lower levels in other organs. PPAR*γ* is highly expressed in adipose tissues and is present in the colon and lymphoid organs. PPAR*β*/*δ* is expressed ubiquitously, but its levels may vary considerably [42, 43].

Mechanistically, the PPARs also form heterodimers with the RXR and activate transcription by binding to a specific DNA element, termed the peroxisome proliferator response element (PPRE), in the regulatory region of several genes encoding proteins that are involved in lipid metabolism and energy balance. Binding of agonists causes a conformational change that promotes the binding to transcriptional coactivators. Conversely, binding of antagonists induces a conformation that favors the binding of corepressors. Physiologically, PPAR-RXR heterodimers may bind to PPREs in the absence of a ligand, although the transcriptional activation depends on the ligand-bound PPAR-RXR [44, 45]. The predominant role of these receptors is the transcriptional regulation of enzymes and other proteins involved in energy homeostasis, some of which are in the liver. To explain their possible action in the development and treatment of NAFLD, a brief description of each PPAR follows [46, 47].

In the liver, PPAR*α* promotes fatty acid oxidation. It is the target for the hypolipidemic fibrates, such as fenofibrate, clofibrate, and gemfibrozil, which are used in the treatment of hypertriglyceridemia [48].

The role of PPAR*α* in hepatic fatty acid metabolism is especially prominent during fasting. In fasted PPAR*α*-null mice, its absence is associated with pronounced hepatic steatosis, decreased levels of plasma glucose and ketone bodies, and elevated plasma free fatty acids levels, and hypothermia. These severe metabolic disturbances are the result of the decreased expression of many genes involved in hepatic lipid metabolism. The PPAR*α* target genes are those for acyl CoA oxidase (ACO-OX), acyl CoA synthase (ACS), enoyl-CoA hydratase, malic enzyme, HMG CoA synthase, mitochondrial enzymes, liver-fatty-acid-binding protein, and fatty acid transport protein. PPAR*α* can also regulate other genes such as LPL, which is involved in the degradation of triglycerides, and APOA1 and APOCIII, which are both downregulated by PPAR*α* [49–55] (Figure 2).

Whereas PPAR*α* controls lipid catabolism and homeostasis in the liver, PPAR*γ* promotes the storage of lipids in adipose tissues and plays a pivotal role in adipocyte differentiation. It is a target of the insulin-sensitizing thiazolidinediones. Despite its relatively low expression levels in healthy liver, PPAR*γ* is critical for the development of NAFLD [56].

In the liver, PPAR*β*/*δ* is protective against liver toxicity induced by environmental chemicals, possibly by downregulating the expression of proinflammatory genes. PPAR*β*/*δ* regulates glucose utilization and lipoprotein metabolism by promoting reverse cholesterol transport [57–60]. PPARs appear to be targets for the treatment of metabolic disorders. PPAR*α* and PPAR*γ* are already therapeutic targets for the treatment of hypertriglyceridemia and insulin resistance, respectively, disorders that relate directly to the progress of NAFLD. The discovery of more pathways may provide new treatments for hepatopathies.

### 5.3. Farnesoid X Receptor

The farnesoid X receptor (FXR), a member of the NR superfamily, has a typical NR structure and contains a hydrophobic pocket that accommodates lipophilic molecules such as bile acids [61]. Its gene is located on chromosome 12, and it is expressed predominantly in the liver, gut, kidneys, and adrenals and at lower levels in white adipose tissue [62, 63]. The FXR binds to specific response elements as a heterodimer with the RXR, although it has also been reported to bind DNA as a monomer [28, 64]. The main physiological role of the FXR is to act as a bile acid sensor in the enterohepatic tissues. FXR activation regulates the expression of various transport proteins and biosynthetic enzymes crucial to the physiological maintenance of bile acids and lipid and carbohydrate metabolism.

Bile acids bind to and activate this NR. The order of potency of FXR binding to bile acids is chenodeoxycholic acid > lithocholic acid = deoxycholic acid > cholic acid [65, 66].

In addition to their well-established roles in bile acid metabolism, recent data have demonstrated that activation of the FXR is also implicated in lipid metabolism. Activation of the FXR reduces both hepatic lipogenesis and plasma triglyceride and cholesterol levels, induces the genes implicated in lipoprotein metabolism/clearance, and represses hepatic genes involved in the synthesis of triglycerides [67]. The FXR promotes reverse transport of cholesterol by increasing hepatic uptake of HDL cholesterol via two independent mechanisms. The first is FXR-mediated suppression of hepatic lipase expression [68]. Hepatic lipase reduces HDL particle size by hydrolyzing its triglycerides and phospholipids in hepatic sinusoids, which facilitates hepatic uptake of HDL cholesterol. The second mechanism is the induction by the FXR of the expression of the gene for scavenger receptor B1, the HDL uptake transporter in the liver [69].

Activation of the FXR also increases the hepatic expression of receptors such as VLDL receptor and syndecan-1, which are involved in lipoprotein clearance, and increases the expression of ApoC-II, which coactivates lipoprotein lipase (LPL). FXR activation also decreases the expression of proteins such as ApoC-III and Angptl3 [70] that normally function as inhibitors of LPL. Finally, the FXR induces human PPAR*α* [71], an NR that functions to promote fatty acid *β*-oxidation. Taken together, these data suggest that FXR activation lowers plasma triglyceride levels via both repressing SREBP1-c and triglyceride secretion and increasing the clearance of triglyceride-rich lipoproteins from the blood (Figure 2).

In carbohydrate metabolism, activation of the hepatic FXR regulates gluconeogenesis, glycogen synthesis, and insulin sensitivity [72]. The bile acid sensor FXR also has anti-inflammatory properties in the liver and intestine, mainly by interacting with NF-*κ*B signaling. FXR agonists might therefore represent useful agents to reduce inflammation in cells with high FXR expression levels, such as hepatocytes, and to prevent or delay cirrhosis and cancer development in inflammation-driven liver diseases.

These data suggest that FXR activation by its ligands would reduce hepatic steatosis and that such activation may have a beneficial role in NAFLD by decreasing hepatic *de novo* lipogenesis, which constitutes the first “hit” of the disease. Inflammatory processes lead to the development of hepatitis and subsequent liver fibrosis. The hepatic FXR appears to be downregulated during the acute-phase response in rodents in a manner similar to that seen for other NRs such as PPAR*α* and the LXR [73].

### 5.4. The Pregnant X Receptor and Constitutive Androstane Receptor

The pregnane X receptor (PXR) and constitutive androstane receptor (CAR) share some common ligands and have an overlapping target gene pattern. The CAR gene is the product of the NR1I3 gene located on chromosome 1, locus 1q23, whereas hPXR is the product of the NR1I2 gene, which is located on chromosome 3, locus 3q12–q13.3 [74–76]. Like most other NRs, the PXR and CAR have an N-terminal DNA-binding domain and a C-terminal ligand-binding domain. PXR and CAR regulate gene expression by forming heterodimers with the RXR.

The PXR is located in the nucleus and has a low basal activity and is highly activated upon ligand binding [77, 78]. By contrast, in the noninduced state, the CAR resides in the cytoplasm. Compounds that activate the CAR and PXR are structurally very diverse; most are small and are highly lipophilic [79]. The PXR is activated by pregnanes, progesterone, and glucocorticoids [80, 81], whereas the CAR is affected both positively and negatively by androstane metabolites, estrogens, and progesterone [82, 83]. For this reason, in addition to functioning as xenobiotic receptors, the PXR and CAR are thought to be endobiotic receptors that influence physiology and diseases [84, 85].

For example, several studies have shown that the PXR induces lipogenesis in a SREBP-independent manner. Lipid accumulation and marked hepatic steatosis in PXR-transgenic mice are associated with increased expression of the fatty acid translocase CD36 (also called FAT) and several accessory lipogenic enzymes, such as SCD-1 and long-chain free fatty acid elongase. CD36, a multiligand scavenger receptor present on the surface of a number of cell types, may contribute to hepatic steatosis by facilitating the high-affinity uptake of fatty acids from the circulation [86]. The CD36 level in the liver correlates with hepatic triglyceride storage and secretion, suggesting that CD36 plays a causative role in the pathogenesis of hepatic steatosis [87]. PXR may also promote hepatic steatosis by increasing the expression of CD36 directly or indirectly through the PXR-mediated activation of PPAR*γ* [86].

**Figure 3:**
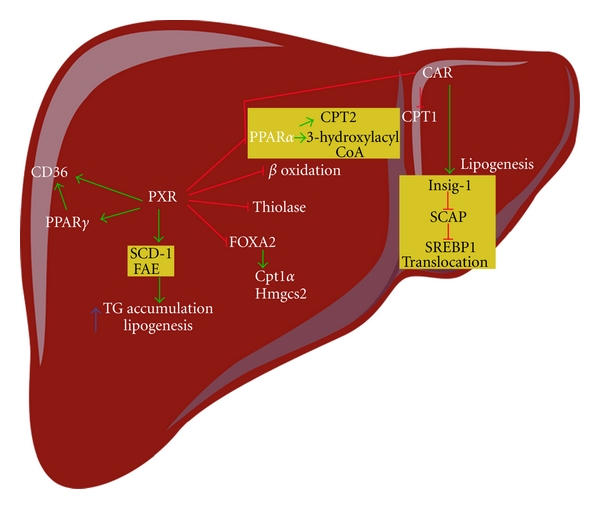
Activation of the PXR induces lipogenesis and inhibits fatty acid *β*-oxidation. The PXR induces lipogenesis through activation of CD36, PPAR*γ*, SCD1, and FAE gene expression. The PXR inhibits fatty acid *β*-oxidation through its suppression of PPAR*α* and thiolase gene expression. In addition, PXR binds to FoxA2, a key regulator of *β*-oxidation, and inhibits FoxA1-mediated activation of Cpt1a and Hmgcs2 gene expression. CAR activation inhibits lipogenesis by inducing Insig-1, a protein that plays a role in SREBP-mediated regulation of lipogenic genes. Insig proteins bind and trap SCAP, retaining it in the ER and preventing it from escorting SREBPs to the site of proteolytic activation in the Golgi complex (not shown). SREBPs are cleaved by two proteases in the Golgi complex, and the bHLH-Zip domain of SREBPs transfers from the membrane to the nucleus to bind the sterol response elements in the promoter region of the target genes (not shown). CAR inhibits fatty acid *β*-oxidation. CAR competes with PPAR*α* for its binding site in the 3-hydroxyacyl CoA dehydrogenase gene promoter. Activation of CAR also decreases the expression of Cpt1, a rate-limiting enzyme of *β*-oxidation. Arrows and stop bars indicate positive regulation or activation and negative regulation or repression, respectively. Cpt1a: carnitine palmitoyltransferase 1a; FAE: long-chain free fatty acid elongase; FoxA2: forkhead box factor A2; Hmgcs2: mitochondrial 3-hydroxy-3-methylglutaryl CoA synthase 2; PPAR: peroxisome proliferator-activated receptor; SCAP: SREBP cleavage-activating protein; SCD1: stearoyl CoA desaturase 1; SREBP: sterol regulatory-element binding protein.

Interestingly, an independent study showed that hepatic triglyceride level decreases temporarily after short-term (10-hour) activation of the PXR [88]. PXR activation is also associated with upregulation of PPAR*γ*, a positive regulator of CD36 and a master regulator of adipogenesis [89]. PXR activation is also associated with suppression of several genes involved in fatty acid *β*-oxidation, such as PPAR*α* and thiolase [90]. A study by Nakamura and colleagues showed that PXR represses *β*-oxidation-related genes such as carnitine palmitoyltransferase 1a (Cpt1a) and mitochondrial 3-hydroxy-3-methylglutaryl CoA synthase 2 (Hmgcs2) through crosstalk with the insulin-responsive forkhead box factor A2 (FoxA2) (Figure 3).

 Activation of the CAR might suppress lipid metabolism and lower serum triglyceride levels by reducing the level of SREBP-1, a master regulator of lipid metabolism. The inhibitory effects of the CAR on lipid metabolism might also be attributed to induction of Insig-1, a protein with antilipogenic properties [88].

The CAR interacts with PPAR*α* during fasting and has been reported to interfere with fatty acid metabolism by binding to DNA elements overlapping with the PPAR*α*-binding site in the promoter region of 3-hydroxyacyl CoA dehydrogenase, an important enzyme in peroxisomal fatty acid *β*-oxidation [91] (Figure 3).

Finally, other studies indicate that the CAR might be involved in the pathogenesis of NASH [92] by regulating the response of serum triglyceride level to metabolic stress [93]. The overlap of the activation of endogenous lipids by the CAR and PXR suggests a functional connection between these receptors in liver physiology. This knowledge might be useful in the development of new treatments to limit or prevent the pathogenesis of NAFLD by developing agonists or antagonists to prevent or lessen lipid accumulation within the liver parenchyma.

## 6. Conclusion

NAFLD encompasses a spectrum of conditions characterized histologically by hepatic steatosis ranging from simple fatty liver to NASH cirrhosis and HCC [4].

NRs control fatty acid transport from peripheral adipose tissue to the liver and regulate several critical metabolic steps involved in the pathogenesis of NAFLD, including fat storage, export, uptake, oxidation, and lipolysis [94]. The discovery that many ligands activate the whole family of NRs (FXR, LXR, PPARs, PXR, and CAR) and their possible interconnected mechanisms that control lipid metabolism suggests the possibility of developing novel therapies for the treatment of NAFLD. The LXR and PXR regulate several metabolically relevant pathways and clusters of genes that lead to hepatic lipogenesis and might be directly related to the pathogenesis of liver diseases. The FXR, PPAR*α*, and CAR are activated by ligands to orchestrate a broad range of lipolytic activities. These might become future candidates for drugs designed to target metabolic liver disorders.

## Figures and Tables

**Table 1:** Nuclear receptors in hepatic lipid metabolism.

RXR partner	Ligands	Official name	Role in hepatic lipid metabolism
LXR*α*	Oxysterols (22(R)-hydroxycholesterol, 24(S)-hydroxycholesterol, 24(S),25-epoxycholesterol, 27-hydroxycholesterol) and fatty acids	NR1H3	(i) Increases fatty acid synthesis, TG level, HDL level, cholesterol secretion
			(ii) Upregulation of SREBP-c{FASACCSCD1
			(iii) Upregulation of ChREBP, Angptl3
			(iv) Downregulation of ApoA-V

PPAR*α*	Fatty acids, fibrates, statins, eicosanoids, and leukotrienes	NR1C1	(i) Promotes fatty acid oxidation (by lipoprotein lipase activation)
			(ii) Improves insulin resistance
			(iii) Suppression: acyl CoA oxidase (ACO-OX), acyl CoA synthase (ACS), enoyl-CoA hydratase, malic enzyme, HMG-CoA synthase, mitochondrial enzymes, APOA1 and APOCIII

FXR	Bile acids, pregnadiene, and fexaramine	NR1H4	(i) Induces lipoprotein metabolism genes/clearance represses hepatic genes involved in the synthesis of TG
			(ii) Induces human PPAR*α*
			(iii) Increases hepatic expression of receptors VLDL
			(iv) Reduces: hepatic lipogenesis and plasma triglyceride and cholesterol levels
			(v) Decreases expression of proteins apoC-III and Angptl3 (inhibitors of LPL)

PXR	Pregnanes, progesterone, and glucocorticoids, LCA, xenobiotics/drugs, rifampicin	NR1I2	(i) Induces lipogenesis by increasing expression of the fatty acid translocase CD36, SCD-1, and long-chain free fatty acid elongase
			(ii) Suppression of several genes involved in fatty acid *β*-oxidation (PPAR*α*, thiolase, carnitine palmitoyltransferase 1a (Cpt1a), and mitochondrial 3-hydroxy-3-methylglutaryl CoA synthase 2 (Hmgcs2))

CAR	Androstane metabolites, estrogens, progesterone, and xenobiotics	NR1I3	(i) Induction of Insig-1, a protein with antilipogenic
			properties
			(ii) Interacts with PPAR*α* during fasting
			(iii) Suppresses lipid metabolism and lowers serum triglyceride level by reducing SREBP-1 level
